# Children’s Medicines in Tanzania: A National Survey of Administration Practices and Preferences

**DOI:** 10.1371/journal.pone.0058303

**Published:** 2013-03-06

**Authors:** Lisa V. Adams, Sienna R. Craig, Elia John Mmbaga, Helga Naburi, Timothy Lahey, Cameron T. Nutt, Rodrick Kisenge, Gary J. Noel, Stephen P. Spielberg

**Affiliations:** 1 Audrey and Theodor Geisel School of Medicine at Dartmouth, Hanover, New Hampshire, United States of America; 2 Dartmouth College, Hanover, New Hampshire, United States of America; 3 Muhimbili University of Health and Allied Sciences, Dar es Salaam, Tanzania; 4 Dartmouth Center for Health Care Delivery Science, Hanover, New Hampshire, United States of America; 5 Institute for Pediatric Innovation, Cambridge, Massachusetts, United States of America; 6 Weill Cornell Medical College, New York, New York, United States of America; 7 AstraZeneca Pharmaceuticals, Waltham, Massachusetts, United States of America; 8 United States Food and Drug Administration, Washington, D.C., United States of America; Nottingham University, United Kingdom

## Abstract

**Objective:**

The dearth of age-appropriate formulations of many medicines for children poses a major challenge to pediatric therapeutic practice, adherence, and health care delivery worldwide. We provide information on current administration practices of pediatric medicines and describe key stakeholder preferences for new formulation characteristics.

**Patients and Methods:**

We surveyed children aged 6–12 years, parents/caregivers over age 18 with children under age 12, and healthcare workers in 10 regions of Tanzania to determine current pediatric medicine prescription and administration practices as well as preferences for new formulations. Analyses were stratified by setting, pediatric age group, parent/caregiver education, and healthcare worker cadre.

**Results:**

Complete data were available for 206 children, 202 parents/caregivers, and 202 healthcare workers. Swallowing oral solid dosage forms whole or crushing/dissolving them and mixing with water were the two most frequently reported methods of administration. Children frequently reported disliking medication taste, and many had vomited doses. Healthcare workers reported medicine availability most significantly influences prescribing practices. Most parents/caregivers and children prefer sweet-tasting medicine. Parents/caregivers and healthcare workers prefer oral liquid dosage forms for young children, and had similar thresholds for the maximum number of oral solid dosage forms children at different ages can take.

**Conclusions:**

There are many impediments to acceptable and accurate administration of medicines to children. Current practices are associated with poor tolerability and the potential for under- or over-dosing. Children, parents/caregivers, and healthcare workers in Tanzania have clear preferences for tastes and formulations, which should inform the development, manufacturing, and marketing of pediatric medications for resource-limited settings.

## Introduction

Nearly seven million children under the age of five die every year, many from treatable conditions for which we lack adequate pediatric medicine formulations. [1] As a result, healthcare workers often report using adult formulations for the treatment of children with diseases such as HIV/AIDS, tuberculosis, and malaria [2], which can lead to reduced efficacy (due to under-dosing) and/or toxicity (due to over-dosing) [3]. Furthermore, information on how best to prepare and administer such drug formulations for children is often lacking in both resource-limited and resource-rich settings [2], [4].

While the 2007 World Health Organization’s Promoting Safety of Medicines for Children booklet and the establishment of the United Nations Commission on Life-Saving Commodities for Women and Children in 2012 have been major steps towards addressing some of these issues, [2], [5]–[9], medication formulations, including qualitative features of form and taste, can affect acceptance and the likelihood of effective administration to pediatric patients. However, information on how best to prepare and administer drug formulations for children is often lacking and research efforts in this area have not been well coordinated [10]–[12].

Certain practical considerations can heavily impact the suitability of pediatric formulations in resource-limited settings. Key drivers of the proper formulation of pediatric medications include parent/caregiver, healthcare worker, and patient preferences for medicine appearance, form, and basic taste (i.e., sweet, sour, bitter) as well as refrigeration requirements, ease of transport, and flavors that correspond to culturally appropriate and recognized food or drink. Yet these factors remain under-studied, especially in resource-limited settings [10]–[14]. Given the critical role that home administration habits, medicine palatability and formulation preferences play in pediatric medication adherence, it is important to understand the practices of parents/caregivers and to engage them in efforts to promote rational use and to improve administration options. [15].

To address these questions, we conducted a cross-sectional survey of parents/caregivers, children, and healthcare workers in rural and urban Tanzania about current administration practices and formulation preferences to guide the development of pediatric medicines and better meet the actual needs of children in resource-limited settings.

## Patients and Methods

### Ethics Statement

Institutional review boards and ethics committees of the World Health Organization (WHO), Dartmouth College, Muhimbili University of Health and Allied Sciences and the National Institute for Medical Research in Tanzania approved this study including the consent procedure. In addition, we obtained permission to conduct surveys from regional authorities in Tanzania. All adult participants provided written informed consent and, due to age and variable writing ability, all child participants provided verbal consent before participation. Verbal consent was obtained by reading an age-appropriate consent form and documenting the child’s verbal agreement on the form. Parental/guardian written informed consent was also obtained for all participating children.

### Study Population and Design

We conducted a cross-sectional survey of a nationwide representative sample of parents/caregivers, children and healthcare workers in Tanzania. We chose Tanzania as the study site based on its ability to represent the East African region and the presence of an existing research collaboration between the authors’ institutions.

Tanzania is divided into four zones, each containing three to six administrative regions. Two representative regions from each zone were selected based on their size, location, economic activities and culture. In addition, two regions from the major urban center of Dar es Salaam and the socioeconomically and culturally distinct island of Zanzibar were also selected, making a total of 10 regions sampled. One urban and one rural district per region were selected using the National Bureau of Statistics census enumerated areas. Rural and urban designations were based on the 2002 census geographical definition, which considers such factors as housing quality, access to services including health care, and poverty levels.

We interviewed a random sample of parents/caregivers of children under age 12 who themselves were aged 18 years or older; healthcare workers who were medical officers, clinical officers, nurses or pharmacists registered with the Ministry of Health; and children between the ages of 6 and 12 years. Given the descriptive objectives of the study, an enrollment target of 200 participants for each cohort was determined to provide a representative national sample.

### Survey Tools and Sampling Methods

Prior to administration, surveys for children, parents/caregivers and healthcare workers were vetted by international pediatric pharmacologists, pediatricians, Tanzanian parents, and medical anthropologists in collaboration with WHO advisors. Surveys were translated into Swahili, and back-translated into English to ensure accuracy. The survey tools consisted of a combination of closed-ended and open-ended questions, with the latter allowing for free text responses. Closed-ended questions were asked without prompting responses, which were then categorized by the interviewers into pre-determined groupings. The three surveys are provided in the supporting information section as Supplement S1 (Parent/Caregiver Survey), Supplement S2 (Healthcare Worker Survey) and Supplement S3 (Child Survey).

We administered our surveys in urban and rural districts within each of 10 regions of Tanzania using a simple random sampling (ballot) method. In the selected district, the resident registry of households, which is regularly updated, was obtained from the ward office. A systematic sampling method was applied to a list of those households with children between 6 and 12 years with the list used as a sampling frame for calculating the sampling interval. The first household on the list was randomly selected using the ballot method and then the sampling interval was applied to reach the target of 200 parents/caregivers and children.

A list of healthcare workers was obtained from the district health authority in each selected district and a random sample of health facilities was selected by the ballot method. After determining the number to be sampled from a health facility, we randomly selected names to recruit from the list of healthcare workers at that facility.

To ensure that families who utilize the health system were included, approximately 25% of the parents/caregivers and children were randomly enrolled at health centers as opposed to residences. The proportion sampled at a specific facility was calculated using the average number of patient visits per day and a sampling interval to provide a reasonable capture of families currently receiving care at that site. Participants were recruited using the sampling interval as they presented at the facility registration desk. Interviews were conducted at health facilities, pharmacy shops (both formally licensed and informal drug dispensers), and in individuals’ homes. In no household were both a parent/caregiver and a child enrolled in the study.

### Study Implementation

Twelve interviewers were selected following advertisement at local universities. Each interview pair was balanced for gender, and regional and disciplinary background (medicine or social science). Muhimbili University of Health and Allied Sciences faculty trained the interviewers in data collection methods and research ethics. A pilot study was conducted 15–16 March 2010 in urban and peri-urban Dar es Salaam, and data collection was conducted between 17 May 2010 and 6 July 2010.

### Data Management and Analysis

Data were reviewed daily in the field for quality. Inconsistent or incomplete responses were followed up with return visits and/or phone calls for data validation. Data were double-entered with unique ID numbers into Access databases and then exported to PASWStatistics (version 18.0, IBM SPSS Inc., Chicago, IL) and Microsoft Excel (version 12.3.0, Microsoft Corporation). To perform the stratified analyses, we imported the data into STATA software (version 9.0, StataCorp, College Station, TX). We used the Kruskal-Wallis or Mann-Whitney *U* tests as appropriate for statistical comparisons with a p-value significance threshold of 0.05.

The three cohorts were stratified into subgroups by variables expected to impact their responses. Parents/caregivers were stratified by setting (defined as urban versus rural) and level of education (defined as having completed less than a primary school education, completed only primary school, and completed more than primary school). Children were stratified by setting and age group. Healthcare workers were stratified by setting and professional cadre. In the latter case, we selected the two largest cadres represented, general practitioners (a category that includes physicians without postgraduate training and non-physician providers such as clinical officers) and registered nurses.

## Results

We collected complete data on 206 children, 202 parents/caregivers, and 202 healthcare workers (Table 1). Overall, balanced distributions by age, setting, and gender were obtained for each group, with the exception that a majority of parents/caregivers was female.

**Table 1:** Survey respondent demographics.

	Parents/caregivers	Children	Healthcare Workers
	(N = 202)	(N = 206)	(N = 202)
Female	177 (88%)	103 (50%)	111 (55%)
Median age (Range)	30 (18–55 years)	10 (6–12 years)	32 (19–57 years)
Urban	105 (52%)	104 (51%)	102 (51%)

The majority of the parent/caregiver households surveyed had a refrigerator or a way to keep medications cold (110 [54.5%]) and most had access to a water tap (142 [70.3%]).

Few parents/caregivers were highly educated: 31 (15.5%) had completed less than a primary school education, 127 (63.5%) had completed primary school only, and 42 (21.0%) had completed more than primary school. The majority of healthcare workers self-identified as general practitioners (52 [25.7%]) or nurses (79 [39.1%]), with smaller proportions of assistant medical officers (31 [15.3%]), dispensary officers (17 [8.4%]), pharmacists (13 [6.4%]), and pediatricians (10 [5.0%]).

### Administration Practices

We asked parents/caregivers about medicine administration for a recent illness episode of one of their children. Oral solid dosage forms (which can include various forms of tablets or capsules), hereafter referred to by the commonly used term “pills”, were the most common formulation administered (113 [72.0%]). The most common methods used by parents/caregivers to administer pills to their children and their experiences with tolerability are summarized in Table 2. Swallowing whole pills or crushing/dissolving pills (including opening and emptying capsule contents) and mixing with water were the two most frequently reported methods of administration. In open-ended questioning about how medicines were administered, parents/caregivers did not mention any other liquids or food items that were used to mix with or dissolve a crushed or broken pill. Qualitative data revealed that parents/caregivers have had to give various fractions, including one-half, one-third, or three-quarters, of a pill or crushed pill powder to their children. A large majority of parents/caregivers (163 [80.7%]) had experienced some problem in the past with giving their children medicines. Parents/caregivers commonly reported children disliking the taste of or vomiting their medicines. When stratified by urban versus rural setting, there were no significant differences in parent/caregiver method of pill administration (P = 0.13) or water source (P = 0.34). Nearly all healthcare workers (192 [95.0%]) reported that vomiting is the main medication administration problem they hear of from parents/caregivers.

**Table 2:** Acute illness medicine administration practices of parents/caregivers.

***Method of Administration***	
Had child swallow whole pill	48/173 (28%)
Had child swallow crushed/broken pill	16/173 (9%)
Had child swallow dry powder from crushed pill	5/173 (3%)
Had child drink crushed/dissolved pill mixed with water	59/173 (34%)
Other method, or unspecified	45/173 (26%)
***Source of water***	
Tap*	45/95 (47%)
Well**	11/95 (12%)
Bottled water or boiled water	39/95 (41%)
Method used to crush pill	
Between 2 spoons	45/74 (61%)
With glass bottle between sheets of paper	29/74 (39%)
***Tolerability***	
Parent/caregiver reported child vomited medicines	49/202 (24%)
Parent/caregiver reported child did not complete all medicines	17/202 (8%)
Child disliked the taste of some medicines	76/202 (38%)

* The location of the tap (village or household) was not specified.

** Depth of the well was not indicated so this may represent a spring source, or deep or shallow well.

### Formulation Preferences

We asked parents/caregivers and children how medicines for children *should* taste. Their responses–overall and stratified by parent/caregiver education level, and child age group–are shown in Table 3. The majority of respondents in every subgroup preferred sweet tasting medicines for children. This finding persisted when the data were stratified by setting. When compared across parent/caregiver education level, there was a statistically significant greater preference for sweet medicines among respondents with more than a primary school education, whereas those with less than a primary school education were less likely to state a preference (P<0.001). Although both child age groups preferred sweet tasting medicines, 100% of children in the 6–8 years age group stated this preference (P = 0.02). There were no statistically significant differences between urban and rural subsets of parents/caregivers or children.

**Table 3:** Parents/caregivers’ and children’s preferred tastes for children’s medicines, stratified by parent/caregiver education level and child age.

	Parent/Caregiver Preferences				Children’s Preferences		
	*Total*	*Less than primary*	*Primary education*	*More than primary*	*Total*	*Age 6–8*	*Age 9–12*
	*(N = 194)*	*(N = 29)*	*(N = 123)*	*(N = 41)*	*(N = 204)*	*(N = 46)*	*(N = 158)*
Sweet	155	14	103	37	186	46	140
	(80%)	(48%)	(84%)	(90%)	(91%)	(100%)	(89%)
Bitter	2	0	2	0	8	0	8
	(1%)	(0%)	(2%)	(0%)	(4%)	(0%)	(5%)
No taste	13	3	9	1	3	0	3
	(7%)	(10%)	(7%)	(2%)	(2%)	(0%)	(2%)
No	24	12	9	3	7	0	7
preference	(12%)	(41%)	(7%)	(8%)	(3%)	(0%)	(4%)

A total of 170 (82.5%) children said they like the taste of some medicines more than others. Children’s reasons for these preferences included medication sweetness (103 [59.5%]), whereas some mentioned specific flavors such as banana, mango, orange, and pineapple. Among the majority of parents/caregivers who reported their children preferred some medicines to others (131 [64.8%]), most (88 [67.2%]) said they preferred sweet flavored medicines and in unprompted, open-ended responses listed a similar selection of specific example flavors such as banana, orange, pineapple, and Orange Fanta® (a soda commonly available in Tanzania).

We asked parents/caregivers about their preferred formulations for children by age group (Table 4). The majority said they prefer to give newborns and infants oral liquid dosage forms (which may include syrups, elixirs, and suspensions), hereafter referred to by the commonly used term “syrups”. While syrups were still the most commonly preferred formulation for a toddler, nearly one-quarter of parent/caregiver respondents preferred to crush pills and mix with water for this age group. The majority of parents/caregivers were divided between preferring crushed/dissolved pills and syrups for children of preschool age (2 to 6 years). Most said they would prefer to give pills to children in primary school (age 6 to 12 years). There were no statistically significant differences in these medication formulation preferences between the parent/caregiver groups when compared by rural versus urban setting or respondent education level.

**Table 4:** Total parent/caregiver medicine formulation preferences for different child age groups.

Age Group	As a Syrup	Crushed/Dissolved Pills with Fluid	Would not give medicineto child this age	Chewable Pill	Swallow Pills
Newborn (N = 185)	178	2	5	N/A	N/A
	(96%)	(1%)	(3%)		
Infant (1–6 mos	177	11	2	N/A	N/A
old) (N = 190)	(93%)	(6%)	(1%)		
Toddler (1 year	147	45	0	0	2
old) (N = 194)	(76%)	(23%)	(0%)	(0%)	(1%)
Preschooler (2–6	59	93	0	7	36
years old) (N = 195)	(30%)	(48%)	(0%)	(4%)	(19%)
Primary schooler	9	17	0	4	165
(6–12 years old)	(5%)	(9%)	(0%)	(2%)	(85%)
(N = 195)					

Note: columns occasionally don’t sum exactly to 100% because of rounding.

We asked parents/caregivers and healthcare workers about the maximum number of pills they thought a child at different ages should take at one time and per day (Table 5). There was generally good agreement between parents/caregivers and healthcare workers for the most commonly selected maximum pill number. In most instances, the maximum numbers selected by healthcare workers were the same or lower than those selected by parents/caregivers.

**Table 5:** Maximum number of pills parents/caregivers or healthcare workers think children should take at one time or per day.

	Maximum pills at one time	
	*Parents/caregivers*	*Healthcare Workers*
Child with teeth	1 pill (152/196, 78%)	1 pill (123/133, 93%)
Child in early years of	1 pill (85/198, 43%)	1 pill (108/192, 56%)
primary school	2 pills (30/198, 15%)*	
Child in last years of primary school	2 pills (104/198, 53%)	2 pills (101/192, 53%)
	**Maximum pills per day**	
	***Parents/caregivers***	***Healthcare Workers***
Child with teeth	3 pills (89/198, 45%)	3 pills (81/161, 50%)
	2 pills (40/198, 20%)	
Child in early years of	6 pills (62/198, 31%)	3 pills (85/194, 44%)
primary school	3 pills (59/198, 30%)	6 pills (58/194, 30%)
Child in last years of	6 pills (94/198, 48%)	6 pills (110/198, 56%)
primary school	9 pills (25/198, 13%)	

* If leading choice represented less than 50% of interviewees, second most popular response was included.

When stratified by parent/caregiver education level, those with the least education preferred lower numbers of pills overall, with the highest percentage preferring only 1 pill and 3 pills per day maximum for children with newly acquired teeth and children in the first years of primary school, respectively. However, we found no statistically significant differences in the preferred median number of pills between parents/caregivers when stratified by educational level.

### Therapeutic Choice

We asked healthcare workers to rank the factors that impact their selection of pediatric medicines (Table 6). Overall, availability was consistently the most important factor when prescribing oral medications to infants and children, whereas taste was consistently the least important factor. There were no significant differences between urban or rural healthcare workers’ selection of the most important factor affecting their choice of medicines for children.

**Table 6:** Most important factor influencing healthcare worker prescriptions to children by age group, total.

Age	Availability	Ease of Administration*	Tolerability	Taste
Newborn	110/196	31/196	34/194	11/196
	(56%)	(16%)	(18%)	(6%)
Infant	98/193	45/193	30/193	12/193
(1–6 mo)	(51%)	(23%)	(16%)	(6%)
Toddler	106/195	37/195	33/195	10/195
(1 year)	(54%)	(19%)	(17%)	(5%)
Child	95/194	32/194	29/194	14/194
(2–6 years)	(49%)	(17%)	(15%)	(7%)
Child	86/195	29/195	45/195	13/195
(6–12 years)	(44%)	(15%)	(23%)	(7%)

* Ease of administration includes answers of “easy to give children,” “easy to give,” and “type/easy to give the child”.

## Discussion

Our study constitutes the largest end-user survey of administration practices and preferences for children’s medicines in a resource-limited country to date. Our findings reveal clear administration patterns and shed light on significant formulation-related challenges faced by parents/caregivers, children, and healthcare workers that impede the rational use of medicines in children.

### Manipulating Adult Formulations

The practice of breaking or crushing pills was common among all parents/caregivers surveyed. This suggests that accurate and complete dosing is infrequent, which risks toxicity, reduced efficacy, and drug resistance [16]–[18]. Palatability, too, can be reduced by breaking or crushing pills [16], particularly if the pill’s format and matrix are designed to mask bitter active ingredients [14], [19], [20].

These practices, as well as the administration of whole pills to young children, can often lead to children vomiting doses immediately upon administration. This problem, commonly reported in our survey, should be addressed by the provision of special instructions to parents/caregivers on what to do if a child vomits medicine.

### Liquid Formulations for Young Children

Age group is the main driver of formulation preferences expressed by parents/caregivers and healthcare workers regardless of setting or education level. Both groups of respondents strongly prefer syrups for newborns, infants, and toddlers. Such preferences have been shown in resource-rich settings (S. Spielberg, MD, PhD, Institute for Pediatric Innovation, unpublished data, 2010), but this is the first survey of these patterns in a resource-limited setting. While not unexpected, our confirmation of these findings may support certain marketing or packaging of medicines, such as color-coding of medicine labels and administration instructions by age group.

All respondents expressed an overwhelming preference for syrups over other delivery forms for younger children, yet syrups are significantly more expensive than dispersible tablet formulations across many categories of medicines and require greater investments in temperature control and transportation [21], [22]. In contrast to our findings, a recent study in Zambia and Uganda found that parents/caregivers preferred pills over syrups for antiretroviral therapy for children above age 3 due to the number, weight, difficult transportation and conspicuousness of the bottles, though investigators did not explore the drawbacks of crushing solid formulations (reduced palatability, lower dosing accuracy, and pharmacokinetic changes) in depth [23].

The WHO currently recommends dispersible tablets and granule sachets adjusted for size and strength as the optimal medicine formulation for young children [21]; our data demonstrate that both forms are currently unfamiliar in Tanzania. Nonetheless, parents/caregivers and healthcare workers were accustomed to crushing portions of adult pills and mixing with water to attain a liquid solution. Therefore, a highly desirable option to improve accuracy and acceptability of pediatric medication formulations would be a dispersible tablet that does not require manipulation to attain the proper dose in liquid form, especially if flavored to suit local taste preferences. Because parents/caregivers actually administer medicines to children at the “last mile” of health care delivery and because they strongly prioritize ease of administration and palatability for pediatric medicines, we believe that great potential exists to enlist them as advocates for the development and delivery of dispersible tablet formulations better suited to local contexts.

Mixing medicines with water or using water to help a child swallow a whole pill has important health implications. Access to clean drinking water is not assured in many regions, including Tanzania, where only 53% of households have access to improved drinking water sources (defined as household connection, public standpipe, borehole, protected dug well, protected spring, or rainwater collection) [24]. Of note, more than one-third of parents/caregivers reported using bottled water with medication use – a finding similar across education levels (which is itself a likely proxy for socioeconomic class) and in both rural and urban settings. This unanticipated finding suggests parents/caregivers will invest in clean water when giving medicines to children, which may indicate early community receptivity to and readiness for dispersible table use.

### Pill Burden Preferences

Parents/caregivers and healthcare workers reported similar thresholds for the acceptable maximum number of pills that children at different ages could take at one time and over the course of one day. More highly educated parents/caregivers report higher numbers of pills are acceptable for every age group. This may indicate a higher comfort level with or greater trust in conventional pharmaceuticals, or higher expectations of their children’s ability to accept or tolerate medicines, among parents/caregivers with more education.

Because parents/caregivers are the ones most likely to administer medicines to children, the limits they express should be used to guide maximum pill burdens in the development of new formulations and regimens. Furthermore, healthcare workers should be encouraged to discuss parent/caregiver expectations and concerns when prescribing regimens with high pill burdens to children.

### Healthcare Worker Prescription Practices

Since the healthcare worker cohort of our study was restricted to those who responded that they provide outpatient care to children, it is notable that relatively few pediatricians and no medical officers were interviewed. Our results suggest that in Tanzania, nurses, general practitioners (including non-physicians), and assistant medical officers provide the majority of primary care to children. Notably, the WHO reports that Tanzania’s public sector physician density is among the lowest of any country in the world [24]. Information about pediatric medicines and other educational efforts should thus be developed with the knowledge that the first-line facility-based healthcare workers are from the cadres with the lowest levels of formal education and without any specialized pediatric training.

Availability was the main determinant of healthcare worker choices for prescription. Problems with availability of children’s medicines at health facilities, especially in forms suitable for newborns and infants, are well documented in low-income countries [25]. One limitation in interpreting this result is the potentially ambiguous meaning of “availability”. Without a more specific definition, some may have interpreted this as “available in their facility”, or “generally available in outside pharmacies in their town”, or “available to parents/caregivers because of reasonable cost” (e.g., accessible). Cost was included as a possible response but was selected at a much lower frequency. It is unclear how many respondents were considering cost in their choice of the more general concept of “availability”. Taste was a much lower priority for healthcare workers than for parents/caregivers and children themselves, a discrepancy that is to some degree inevitable but which nonetheless should beget continued work on healthcare worker sensitization around factors potentially impacting medication adherence among children.

We acknowledge our study’s limitations. First, we did not ask directly about non-conventional or traditional medicine use. In response to more general care-seeking questions, reports of their use were rare, although our qualitative data and observations during fieldwork often revealed that a range of traditional medicines and practitioners are used more often than was formally reported [unpublished data]. Second, few parents/caregivers reported any chronic or longstanding illnesses in their children, making our findings most applicable to short-term medicine administration and begging a similar survey regarding chronic medicine (i.e. antiretroviral and anti-tuberculosis drugs or opportunistic infection prophylactics) usage preferences among a population with similar demographic characteristics be conducted. Third, we recognize that urban and rural distinctions (which often fall along a spectrum) are difficult to categorize with a binary variable and that population movement could result in pattern variations. However, the census classification we used provided a consistent and feasible means of categorizing these areas. Finally, we did not ask about the availability of scored tablets (which may provide consistent dosing) or the availability of or preferences for dosing devices for use with liquid formulations. Liquid doses are often measured inaccurately [26], but parent/caregiver education on the use of certain dosing devices has been shown to improve dose accuracy [27].

### Conclusion

There are many obstacles to the accurate and effective administration of medicines to sick children in resource-limited settings. Poor palatability to the point of emesis, the need to prepare and dose medicines by crushing pills, measuring this bitter powder and mixing with water, and the unavailability of safe drinking water are all common barriers to optimal medication administration in rural and urban Tanzania. Since adherence to treatment supports better clinical outcomes and safety, understanding the formulation preferences of parents/caregivers and children is crucial. Any new medicine format will require study, buy-in from practitioners, and field assessment in multiple cultural and socioeconomic contexts. Beyond the identification of and access to essential medicines for children worldwide [28], the provision of affordable and palatable formulations to promote the rational use of pharmaceuticals in sick children around the world should remain a major goal of global health development efforts.

## Supporting Information

Supplement S1 **Children’s Medicines Practices and Preferences Survey for Parents/Caregivers.** This supplemental item is the survey instrument we used to interview parents/caregivers in Tanzania about their administration practices of and preferences for children’s medicines.
(DOC)

Supplement S2 **Children’s Medicines Practices and Preferences Survey for Healthcare Workers.** This supplemental item is the survey instrument we used to interview healthcare workers in Tanzania about their prescribing practices and preferences for children’s medicines.
(DOC)

Supplement S3 **Children’s Medicines Practices and Preferences Survey for Children.** This supplemental item is the survey instrument we used to interview children in Tanzania about their experience in receiving and preferences for children’s medicines.
(DOC)
