# Supplementary material for: Children’s Medicines in Tanzania: A National Survey of Administration Practices and Preferences
Source: PLoS One. 2013 Mar 6;8(3):e58303. doi: 10.1371/journal.pone.0058303 (PMC3590153; doi:10.1371/journal.pone.0058303)
Supplement: Supplement S1 — Children’s Medicines Practices and Preferences Survey for Parents/Caregivers. This supplemental item is the survey instrument we used to interview parents/caregivers in Tanzania about their administration practices of and preferences for children’s medicines. (DOC) [file pone.0058303.s001.doc]

**Supplement S1. Children’s Medicines Practices and Preferences Survey for Parents/Caregivers**

1. **Eligibility for Participation in the Parent/Caregiver Survey:**
   1. Is the interviewee a parent or primary caregiver over the age of 18 for a child under age 12?

Yes  No

- 1. Has informed consent been obtained? Yes  No

***If Yes checked for both, then proceed. If not, then either correct or stop.***

1. **Demographic Information:**
   1. Location: Region_______________________
      1. District______________________
      2. Ward_________________________________
      3. Village______________________
   2. Gender of Interviewee: M  F
   3. What year were you born? ____________ (or if unknown approximate age___________)
   4. How many people live in your household (include yourself)? ___________
      1. How many children under 12 years of age [in primary school or younger] are living in your household? _____________

Please tell me their first names starting with the oldest [fill in first names, genders and ages now]:

- - 1. Name________________________ Gender_____ Age________ Weight________
    2. Name________________________ Gender_____ Age________ Weight________
    3. Name________________________ Gender_____ Age________ Weight________
    4. Name________________________ Gender_____ Age________ Weight________
    5. Name________________________ Gender_____ Age________ Weight________
    6. Name________________________ Gender_____ Age________ Weight________
  1. Do you know the weight of these child(ren)?  Yes  No [If yes, fill in weights above.]
     1. **If Yes**, *how* do you know? (Check all that apply.)

Have scale at home

Child weighed at health facility

Child weighed on roadside scale

Estimating

Other__________________________________

- 1. Have any children in your household died before they were 5 years old?

Yes  No  Don’t Know

**If yes,** list name, gender, age at death, and cause(s) of death:

- - 1. Name___________________ Gender_____ Age________ Cause(s)______________
    2. Name___________________ Gender_____ Age________ Cause(s)______________
    3. Name___________________ Gender_____ Age________ Cause(s)______________

1. **Childhood Illnesses – Acute Illness Episodes**
   1. Has your child or any of your children ever been sick?  Yes  No  Don’t Know

***If No or Don’t Know, skip to section 5***

- 1. **If Yes**, what are some of the illnesses he/she/they ever had?

_____________________________________________________________________________

- 1. Think about the last time one of your younger children was very sick. What illness or illnesses did your child have? __________________________________________________________
     1. How long did this illness last?

A few days

1-2 weeks

About a month

More than a month

Don’t know

- - 1. What did you do to make your child feel better? ***Check all that apply***.

Took child to health facility

Bought medicines at pharmacy or market and gave at home

Bought/gave traditional herbal medicines

Bought/gave other alternative medicines

Gave special foods or drinks

Nothing

Other, specify________________

Don’t remember

- - 1. If you took your child to a health facility, where did you go first? Did you go anywhere else? ***Mark all that apply, beginning with 1 for the place you went first.***

Public clinic

Public hospital

Pharmacy

Private clinic

Private hospital

Traditional healer

Other, specify_________________

- - 1. Did your child take any medicines for this illness?

Yes  No  Don’t Remember

***If No or Don’t Remember, skip to Section 4.***

- - - 1. **If Yes,** what types were they? ***Check all of the types that apply***.

Injections

Pills

Chewable tablets

Syrups

Suppositories (medicines that are inserted in the rectum)

Herbs or other traditional treatments

Other______________________________

- - - 1. **If your child took medicines,** where did you get these medicines? ***Mark all that apply*.**

Provided by the public health facility

Provided by the private health facility

Bought at Pharmacy or market

Given by a relative or friend

Provided by a traditional healer

Other__________________

Don’t remember

- - - 1. **If your child took medicines,** did you have to pay for any of the medicines?

Yes, all  Yes, some  No, none  Don’t Remember

- - - 1. **If you had to pay for medicines**, how difficult was it for you to pay for these medicines?

Very difficult  Somewhat difficult  Not too difficult  Not difficult at all

Don’t Remember

- - - 1. If you had to pay for medicines, what did you do to come up with the money?_________________________________________________________
      2. **If your child took medicines for this illness**, did **you have to** give medicines taken by mouth to your child?  Yes  No  Don’t Remember

***If no or don’t remember, skip to Section 4***

- - - 1. Who told you to how to give this/these medicine(s)?

Doctor

Nurse

Pharmacist

Family member

Traditional healer

No one

Don’t remember

Other______________________________

- 1. **If you gave your child medicines pills or tablets**, how did s/he take them?

Swallowed the pill or tablet whole

Broke pill or tablet and swallowed

Crushed pill or tablet (whole or pieces) and gave dry powder

Crushed/dissolved whole or piece of pill or tablet and mixed with water

Any other method__________________________________

- - 1. If you crushed the pill or tablet and mixed it with water, what was the source of this water?____________________________
    2. Did you boil this water before you mixed it with the pill or tablet?

Yes  No  Don’t Remember

- - 1. When mixed with pill or tablet, was the water:

Hot  Cold  Room temperature/Warm  Don’t remember

- - 1. If you crushed the pill or tablet before giving it, how did you crush it?

Between 2 spoons

Between 2 pieces of paper and rolling glass or bottle over it

Other______________________________________________

Don’t remember ***[Ask how you would crush a medicine today]***

3.4.1 Can you show me what you did (or would do)?

[***Describe what you see***]_____________________________

- 1. Did your child ever vomit or spit out their medicines after you gave them?

Yes  No  Don’t Know

- - 1. **If Yes**, when did it happen?

Within a few minutes  Within an hour  More than an hour later  Don’t Remember

- - 1. If your child vomited or spit out the medicines, what did you do for that dose of medicine?

Give the medicine again as soon as possible

Skip a dose and give next dose at scheduled time

Stop giving the medicine completely

Nothing

Other

- 1. How long did you give medicines to your child for this illness?

Less than 2 weeks

More than 2 weeks

Don’t remember

- 1. Were there some medicines prescribed by a health care provider or pharmacist that your child did not finish taking?

Yes  No  Don’t Know

3.7.1. If yes, *why*? ________________________________

- 1. Were there any medicines that your child did not like the taste of?  Yes  No  Don’t Know

3.8.1. If yes, *why*?__________________________________

1. **Childhood Illnesses – Chronic Illness Episodes**
   1. Do any of your children have an ongoing illness?  Yes  No  Don’t Know

***If no or Don’t know, skip to section 5***

- - 1. If Yes, list the child and their ongoing illness(es).
       1. Child first name_________________ Ongoing illness(es) ________________
       2. Child first name_________________ Ongoing illness(es) ________________
       3. Child first name_________________ Ongoing illness(es) ________________
       4. Child first name_________________ Ongoing illness(es) ________________

***We would now like to ask you some questions about your child with an ongoing illness. If you have more than one child with an ongoing illness, please provide responses about your youngest child with an ongoing illness. If this child has more than one ongoing illness, please provide responses about the illness you think is most serious.***

- 1. How did you learn your child has this illness?

From a health care provider

From a laboratory test

From a traditional healer

From an alternative medicine practitioner

From a family member

Other___________________________

- 1. When did you learn that your child has this illness?

Within last month

1-3 months ago

4-6 months ago

7-12 months ago

> 12 months ago

Don’t know

- 1. How does your child having an ongoing illness affect you? _____________________________________________________________
  2. How does your child having an ongoing illness affect your family? _________________________________________________________________
  3. Do you regularly give your child any medicines for this illness? ***Check all that apply. If no, then skip to question 4.13***

Injections

Pills

Chewable tablets

Syrups

Suppositories (medicines that are inserted in the rectum)

Traditional / herbal medicines

Other______________________________

- 1. Where do you get these medicines?

Provided by public health facility

Provided by private health facility

Bought at pharmacy or market

Given by a relative or friend

Given by traditional healer

Given by an alternative medicine practitioner

Other__________________

- 1. How difficult was it for you to pay for these medicines?

Very difficult  Somewhat difficult  Not too difficult  Not difficult at all

Don’t Remember

- 1. What did you do to come up with the money? _________________________
  2. Who first told you to how to administer this/these medicine(s)?

Doctor

Nurse

Pharmacist

Family member

Traditional healer or herbalist

No one

Don’t remember

- 1. Do you give pills or tablets regularly to your child for this illness?  Yes  No  Don’t Know
  2. **If yes**, how do you typically give these pills or tablets to your child?

Swallowed the pill or tablet whole

Broke pill or tablet and swallowed

Crushed whole pill or tablet or piece of pill and gave dry powder

Crushed/dissolved whole or piece of pill or tablet and mixed with water

Any other method__________________________________

- - 1. If you crushed the pill or tablet and mixed it with water, what was the source of this water?____________________________
    2. Did you boil this water before you mixed it with the pill or tablet?

Yes  No  Don’t Remember

- - 1. When mixed with pill or tablet, was the water:

Hot  Cold  Room temperature/Warm  Don’t remember

- - 1. If you crushed the pill or tablet before giving it, how did you crush it?

Between 2 spoons

Between 2 pieces of paper and rolling glass or bottle over it

Other______________________________________________

Don’t remember ***[Ask how you would crush a medicine today]***

4.12.4.1. Can you show me what you did (or would do)? _____________________________ [***Describe what you see***]

- 1. Did your child ever vomit or spit out their medicines after you gave them?

Yes  No  Don’t Know

- - 1. **If Yes,** when did it happen?  Within a few minutes  Within an hour  More than an hour later  Don’t Remember
    2. If your child vomited or spit out their medicines, what did you do for that dose of medicine?

Give the medicine again as soon as possible

Skip a dose and give next dose at scheduled time

Stop giving the medicine completely

Nothing

Other___________________________________________

- 1. In an average week, are there some medicines prescribed by a health care provider or pharmacist that your child does not finish taking? Yes  No  Don’t Know

4.14.1 If yes, *why*?___________________________________________

- 1. Are there any medicines that your child does not like the taste of?

Yes  No  Don’t Know

4.15.1 If yes, *why*?__________________________________

- 1. Are there some forms of medicines that you prefer for this child? ***Check all that apply***

Syrups

Pills

Chewable tablets

Injections

Suppositories (medicines that are inserted into the rectum)

Other

4.16.1 *Why* do you prefer this/these forms of medicines? _____________________________

- 1. Are there some forms of medicines that your child seems to prefer?

Syrups

Pills

Chewable tablets

Injections

Suppositories

Other_________________________

1. **Medicines: Use and Access and Expectations**
   1. What are the 2 or 3 most common medicines you give to your child/children?_____________
   2. Have you ever had problems giving your child/children any medicines?

Yes  No  Don’t Know

5.2.1. If Yes, what were the problems?_________________________________

- 1. Were you able to solve these problems?  Yes  No  Don’t Know
     1. If yes, *how*?________________________________________
  2. What do you think medicines for children should taste like?

Sweet

Bitter

No taste

Other ______________

No preference

Don't know

- 1. Are there specific tastes of medicine that your child(ren) seems to prefer?

Yes  No  Don’t Know

5.5.1 If Yes, what are they? ______________________________

- 1. What do you think are the best types of medicines for children? ______________________

5.7.1 *Why*? ___________________________________________________________

- 1. Do you think children and adults can take the same medicines, just in different amounts? Why or why not? _______________________
  2. Are there any medicines you think are harmful for children?  Yes  No  Don’t Know

5.11.1. If yes, which ones? _____________________________

5.11.2 *Why*? _____________________________________________________

- 1. When you bring your child to a health facility do you hope to receive medicines?

Yes  No  Don’t Know

- - 1. If yes, why?___________________________
    2. If no, why not? ________________________
  1. How quickly do you expect a medicine you give to your child to work? _________________
  2. Do you think medicines you give to your child for one illness can sometimes be used to treat a different illness?  Yes  No  Don’t Know

5.11.1. If yes, which medicines can be used for which illnesses?_______________________

- 1. For a baby only a few weeks old, how would you prefer to give that child medicine?

As a syrup

As a pill that you crushed/dissolved and mixed with some liquid

Other____________________

Would not give medicine to a child this age

- 1. For a baby still nursing or taking formula but not yet walking (1 to 6 months old), how would you prefer to give that child medicine?

As a syrup

As a pill that you crushed/dissolved and mixed with some liquid

Other____________________

Would not give medicine to a child this age

- 1. For a child that has some teeth and has just started walking (approximately one year old), how would you prefer to give that child medicine?

As a syrup

As a pill that you crushed/dissolved and mixed with some liquid

A chewable tablet

Other____________________

Would not give medicine to a child this age

- 1. For a child that has its teeth and can talk (2 to 6 years old), how would you prefer to give that child medicine?

As a syrup

As a pill that you crushed/dissolved and mixed with some liquid

A chewable tablet

A pill to swallow

Other____________________

Would not give medicine to a child this age

- 1. For a child who is in primary school (6 to 12 years old), how would you prefer to give that child medicine?

As a syrup

As a pill that you crushed/dissolved and mixed with some liquid

A chewable tablet

A pill to swallow

Other____________________

Would not give medicine to a child this age

***[Show actual pills of four different sizes.]***

- 1. Which of these do you think is the largest pill a child that has its teeth and can talk (2-6 years) could take? ______
     1. How would you give this medicine to a child of this age?_________________
  2. Which of these do you think is the largest pill a child who is in primary school (6-12 years) could take? ____________
     1. How would you give this medicine to a child of this age?_________________
  3. If you would crush the medicine before giving it, how would you crush it?

Between 2 spoons

Between 2 pieces of paper and rolling glass or bottle over it

Other______________________________________________

Don’t know

5.19.1 How you would crush it? ***_____________________________[Describe what you see]***

***[SHOW SIZE "A" PILL]***

- 1. If this pill had the right amount of medicine in it for a baby only a few weeks old, how would you give it?____________

***[Ask to describe exactly: if would crush it, how? If would dissolve it, in what liquid?]***

- 1. [Show size A pill] If this pill had the right amount of medicine in it for a baby that was still nursing but not yet walking (1 to 6 months old) how would you give it?____________

***[Ask to describe exactly: if would crush it, how? If would dissolve it, in what liquid?]***

- 1. If this pill had the right amount of medicine in it for a child that has just started walking (approximately one year old), how would you give it?___________________

***[Ask to describe exactly: if would crush it, how? If would dissolve it, in what liquid?]***

- 1. If this pill had the right amount of medicine in it for a child that has its teeth and can talk (2 to 6 years old), how would you give it? ___________________________

***[Ask to describe exactly: if would crush it, how? If would dissolve it, in what liquid?]***

***[SHOW SIZE "B" PILL]***

- 1. If this pill had the right amount of medicine in it for a baby only a few weeks old, how would you give it?____________

***[Ask to describe exactly: if would crush it, how? If would dissolve it, in what liquid?]***

- 1. If this pill had the right amount of medicine in it for a baby that was still nursing but not yet walking (1 to 6 months old) how would you give it?____________

***[Ask to describe exactly: if would crush it, how? If would dissolve it, in what liquid?]***

- 1. If this pill had the right amount of medicine in it for a child that has just started walking (approximately one year old), how would you give it?___________________

***[Ask to describe exactly: if would crush it, how? If would dissolve it, in what liquid?]***

- 1. If this pill had the right amount of medicine in it for a child that has its teeth and can talk (2 to 6 years old), how would you give it? _________________________________

***[Ask to describe exactly: if would crush it, how? If would dissolve it, in what liquid?]***

***Show a dispersible tablet and describe how it will dissolve easily in liquid and how it is different from other pills.***

- 1. How would you give this kind of pill to a baby?______________________________
  2. What is the maximum number of pills a child will be able to take at one time?

5.29.1. A child with teeth and talking, about 2-3 years old _________

5.29.2. A child in the first years of primary school, about 6-8 years old ________

5.29.3. A child in the last years of primary school, about 9-12 years old ________

- 1. What is the maximum number of pills a child will be able to take in a day?

5.30.1. A child with teeth and talking, about 2-3 years old _________

5.30.2. A child in the first years of primary school, about 6-8 years old ________

5.30.3. A child in the last years of primary school, about 9-12 years old ________

1. **Socioeconomic Indicators and Health Access**
   1. What is the highest grade of school that you completed?

None

Primary school

Secondary School

Institution of higher learning (college or university)

Post-graduate

- 1. **Including yourself**, is anyone in your household currently earning a cash income?

Yes  No  Don’t Know

- - 1. **If Yes**, list member of household and their type of work (for interviewee list as SELF).

Relationship______________________ Type of work_________________________

Relationship______________________ Type of work _________________________

Relationship______________________ Type of work _________________________

Relationship______________________ Type of work _________________________

- 1. How much money do you spend in one day for the needs of your family?________________
     1. Do you or anyone in your family own land (separate from your home)?

Yes  No  Don’t Know

- - 1. Do you or your family own your home?  Yes  No  Don’t Know
  1. What is the source / sources of electricity, if any, in your home?______________________
  2. Do you have a refrigerator or ways to keep food and drinks cold?  Yes  No  Don’t Know
  3. Where do you get your drinking water?

Tap in the home

Tap stand in the neighborhood

Well

River/stream

At a store

From neighbor

Other__________________________________

- 1. What is the level of the health facility nearest your home?

Health outpost

District level facility

Regional level facility

Referral level facility

Don’t know

- 1. What type of health facility is it?

Public facility

Private facility

Don't know

Other ___________________

- 1. How do you usually travel to that facility?

Walk

Bicycle

Private car

Dala dala

Taxi or hired car

Other_________________________

- 1. How much time does it take you to travel to that facility?

Less than 15 minutes

15 minutes – 30 minutes

31 minutes – 1 hour

1 – 2 hours

>2 hours

- 1. If you pay for transportation to the facility, how much does it typically cost?

0-500 T Schillings

501-1,000

1,001-1,500

1,500-3,000

>3,000

Don’t know
