# Supplementary material for: Children’s Medicines in Tanzania: A National Survey of Administration Practices and Preferences
Source: PLoS One. 2013 Mar 6;8(3):e58303. doi: 10.1371/journal.pone.0058303 (PMC3590153; doi:10.1371/journal.pone.0058303)
Supplement: Supplement S2 — Children’s Medicines Practices and Preferences Survey for Healthcare Workers. This supplemental item is the survey instrument we used to interview healthcare workers in Tanzania about their prescribing practices and preferences for children’s medicines. (DOC) [file pone.0058303.s002.doc]

**Supplement S2. Children’s Medicines Practices and Preferences Survey for Healthcare Workers**

1. **Eligibility for Participation in the Health Care Worker Survey:**
   1. Is the interviewee registered in their profession? Yes  No
   2. Has informed consent been obtained? Yes  No

***If Yes checked for both, then proceed. If not, then either correct or stop.***

1. **Health Facility Setting Information:**
   1. Location of Facility: Region: _________________________________
   2. District: _________________________________
   3. Ward_________________________________
   4. Village______________________
   5. Type of facility:  Public Facility  Private Facility Other______________
   6. What is the level of your facility?

Health outpost

District level facility

Regional level facility

Referral level facility

Other __________________________________

Don’t know

- 1. Gender of Interviewee: M  F
  2. What year were you born? ____________ (or if unknown, approximate age_________)
  3. What is your cadre? Pediatrician  general practitioner  clinical officer  Assistant Medical Officer  Nurse  Pharmacist
  4. Do you treat (or provide treatment to) children younger than age 12 in your practice? Yes  No

***If No, STOP and identify another health care worker to interview at this facility.***

- 1. How many children younger than age 12 do you see in a typical week:_________
  2. What are the three most common illnesses you diagnose/treat in children younger than 12 during the course of year? ***Check all that the respondent mentions***.
     1. Malaria
     2. Upper respiratory infections
     3. Lower respiratory infections, such as pneumonia
     4. Diarrheal disease
     5. Urinary tract infections
     6. Skin infections
     7. Schistosomiasis
     8. General or other worms/parasites
     9. Malnutrition
     10. Other __________________________
     11. Other___________________________
  3. What are the three most common oral medicines, excluding vaccines and vitamins, that you prescribe/dispense to children under 12 during the course of a year?

Medicine 1: _________________________________

Medicine 2: ___________________________________

Medicine 3: ___________________________________

- 1. Where do the parents/caregivers obtain medicines that you prescribe/dispense for their children? ***Check all that apply.***

At your facility

At a separate pharmacy

Other__________________

Don’t know

- 1. Do you have a list of medicines that are available and dispensed at your facility?

Yes  No  Don’t remember

***If yes, ask for a copy of this list.***

1. **Current Practices of Prescription and Administration**
   1. When prescribing/dispensing a medicine for a baby only a few weeks old, how do you decide on the dose?

By their age

By their weight

By both their age and weight

Other________________

- - 1. If by their weight, how do you obtain their weight?

I weight them on a scale

I estimate their weight

I ask the child or their parent/caregiver for their child’s weight

Other_________________________________

- 1. When prescribing/dispensing a medicine for a baby still nursing or taking formula but not yet walking (1-6 months) how do you decide on the dose?

By their age

By their weight

By both their age and weight

Other________________

- - 1. If by their weight, how do you obtain their weight?

I weight them on a scale

I estimate their weight

I ask the child or their parent/caregiver for their child’s weight

Other_________________________________

- 1. When prescribing/dispensing a medicine for a child that has just started walking (approx. 1 year) how do you decide on the dose?

By their age

By their weight

By both their age and weight

Other________________

- - 1. If by their weight, how do you obtain their weight?

I weight them on a scale

I estimate their weight

I ask the child or their parent/caregiver for their child’s weight

Other_________________________________

- 1. When prescribing/dispensing a medicine for a child that has its teeth and can talk (2 to 6 years) how do you decide on the dose?

By their age

By their weight

By both their age and weight

Other________________

- - 1. If by their weight, how do you obtain their weight?

I weight them on a scale in my office

I estimate their weight

I ask the child or their parent/caregiver for their child’s weight

Other_________________________________

- 1. When prescribing/dispensing a medicine for a child in primary school (6 to 12 years) how do you decide on dose?

By their age

By their weight

By both their age and weight

Other________________

- - 1. If by their weight, how do you obtain their weight?

I weight them on a scale

I estimate their weight

I ask the child or their parent/caregiver for their child’s weight

Other_________________________________

- 1. At your facility, who typically advises the parent/caregiver how to administer oral medicines to a child under age 12?

Physician

Nurse

Pharmacist at the health care facility

Pharmacist outside of health care facility

No one

Other __________________________

- 1. Have you ever prescribed/dispensed medicine to a child under age 12 that was only available in adult dosage form? Yes  No  Don’t remember
     1. If Yes, which medicines? ______________________________
  2. For the formulations of oral medicines listed below, state if and why you prefer to prescribe/dispense them to children? I prefer to prescribe:
     1. Pills that need to be swallowed
        1. *Why*? ___________________________________
     2. Dissolvable tablets
        1. *Why*? ____________________________________________
     3. Chewable tablets
        1. *Why*? _____________________________________________
     4. Syrups or liquid suspensions
        1. *Why*? ____________________________________
     5. Meltable strips
        1. *Why*? ________________________________________________
     6. Other______________________________
        1. *Why*? _________________________
  3. If more than one medically appropriate option exists, what are the main factors that influence your choice of which oral medicine(s) to prescribe to a baby a few weeks old? ***Please tell us the 3 most important factors***.
     1. Most important______________________
     2. Next most important__________________
     3. Next most important__________________

Factors:

1 = Availability

2 = Price

3 = Formulation / Ease of administration

4 = Lowest side effect profile

5 = Taste

6 = Tolerability

7= Other ______________________________________

- 1. If more than one medically appropriate option exists, what are the main factors that influence your choice of which oral medicine(s) to prescribe to a child that is still nursing or taking formula but not yet walking (1 to 6 months)? ***Please select the 3 most important factors***.
     1. Most important______________________
     2. Next most important__________________
     3. Next most important__________________

Factors:

1 = Availability

2 = Price

3 = Formulation / Ease of administration

4 = Lowest side effect profile

5 = Taste

6 = Tolerability

7= Other ______________________________________

- 1. When more than one medically appropriate option exists, what are the main factors that influence your choice of which oral medicine(s) to prescribe to a child that has just started walking (approx. 1 year old)? ***Please select the 3 most important factors***.
     1. Most important______________________
     2. Next most important__________________
     3. Next most important__________________

Factors:

1 = Availability

2 = Price

3 = Formulation / Ease of administration

4 = Lowest side effect profile

5 = Taste

6 = Tolerability

7= Other ______________________________________

- 1. When more than one medically appropriate option exists, what are the main factors that influence your choice of which oral medicine(s) to prescribe to a child that has its teeth and can talk (2 to 6 years)? ***Please select the 3 most important factors***.
     1. Most important______________________
     2. Next most important__________________
     3. Next most important__________________

Factors:

1 = Availability

2 = Price

3 = Formulation / Ease of administration

4 = Lowest side effect profile

5 = Taste

6 = Tolerability

7= Other ______________________________________

- 1. When more than one medically appropriate option exists, what are the main factors that influence your choice of which oral medicine(s) to prescribe to a child in primary school (6 to 12 years)? ***Please select the 3 most important factors***.
     1. Most important______________________
     2. Next most important__________________
     3. Next most important__________________

Factors:

1 = Availability

2 = Price

3 = Formulation / Ease of administration

4 = Lowest side effect profile

5 = Taste

6 = Tolerability

7= Other ______________________________________

- 1. How important is the formulation of a medicine to you when making a decision about which medicine to prescribe/dispense?

Very important  Somewhat important  Neutral  Not very important

Not at all important

3.14.1 *Why*? ___________________________________________________________________

- 1. Do you give any fixed-dose combination tablets to children under 12?

Yes  No  Don’t know

**If Yes**, what are the advantages to giving fixed-dose combination tablets versus multiple individual medicines to your patients younger than age 12?

- - 1. Advantage #1_________________
    2. Advantage #2__________________
    3. Advantage #3__________________

Advantages:

1 = Usually fewer total number of pills for patient to take at one time

2 = Improves adherence. If so, why?__________________________________

3 = Simpler / less confusing regimen for patients

4 = Other________________________________________

**If Yes**, what are the disadvantages to giving fixed-dose combination tablets versus multiple individual medicines to your patients younger than age 12?

- - 1. Disadvantage #1_________________
    2. Disadvantage #2__________________
    3. Disadvantage #3__________________

Disadvantages:

1 = Some FDCs not in appropriate combination for giving to children

2 = Pills are bigger in size

3 = More difficult to identify causative agent in a drug reaction

4 = More difficult to tailor/individualize regimen to patient’s needs

5 = Other ____________________________________________

- 1. [***Show actual pills of four different sizes.]*** Which of these do you think is the largest pill a child that has its teeth and can talk could take? ______
     1. How would you advise a parent/caregiver to give this medicine to a child of this age?

Swallow the pill or tablet whole

Break pill or tablet and swallow

Crush whole pill or tablet or piece of pill and give dry powder

Crush/dissolve whole or piece of pill or tablet and mix with water

Any other method__________________________________

- - 1. If you would advise to crush the pill or tablet and mix it with water, what should be the source of this water?____________________________
    2. Would you advise to boil this water before mixing it with the pill or tablet?

Yes  No  Don’t Know

- - 1. When mixed with the pill or tablet, should the water be:

Hot  Cold  Room temperature/Warm  Don’t know

- - 1. If you would crush the pill or tablet before giving it, how would you crush it?

Between 2 spoons

Between 2 pieces of paper and rolling glass or bottle over it

Other______________________________________________

- 1. Which do you think is the largest pill a child in primary school could take? ____
     1. How would you advise a parent/caregiver to give this medicine to a child of this age?

Swallow the pill or tablet whole

Break pill or tablet and swallow

Crush whole pill or tablet or piece of pill and give dry powder

Crush/dissolve whole or piece of pill or tablet and mix with water

Any other method__________________________________

- - 1. If you would advise to crush the pill or tablet and mix it with water, what should be the source of this water?____________________________
    2. Would you advise to boil this water before mixing it with the pill or tablet?

Yes  No  Don’t Know

- - 1. When mixed with the pill or tablet, should the water be:

Hot  Cold  Room temperature/Warm  Don’t know

- - 1. If you would crush the pill or tablet before giving it, how would you crush it?

Between 2 spoons

Between 2 pieces of paper and rolling glass or bottle over it

Other______________________________________________

1. **Challenges in Taking Medicines**
   1. Have you ever treated a patient younger than age 12 for an illness that required taking an oral medicine for more than two weeks? Yes  No  Don’t know
      1. **If Yes,** were you aware of problems that the patient had taking their medicine regularly? Yes  No  Don’t know
      2. **If Yes,** what were the problems?____________________________________________
      3. Were they resolved? Yes  No  Don’t know
      4. If resolved, how?_______________________________________________________
   2. Have parents/caregivers ever told you about problems they encounter administering medicines to their children? Yes  No  Don’t know
      1. **If Yes,** what have the parents/caregivers reported? ***Check all that apply***.

vomiting/spitting out medicine

Allergic reaction/rash

Poor adherence/stopped taking medicines

Side-effects from the medicine

Loss of appetite

Other_______________________________

- 1. Have parents/caregivers ever reported that their child vomits after taking their medicine?

Yes  No  Don’t know

- - 1. **If Yes,** what do you advise them to do?_______________________________________
  1. What do you think is the maximum number of pills a child will be able to take at one time?
     1. A child with teeth and talking _________
     2. A child in the first years of primary school ________
     3. A child in the last years of primary school ________
  2. What do you think is the maximum number of pills a child will be able to take in a day?
     1. A child with teeth and talking _________
     2. A child in the first years of primary school ________
     3. A child in the last years of primary school ________
