# Supplementary material for: Children’s Medicines in Tanzania: A National Survey of Administration Practices and Preferences
Source: PLoS One. 2013 Mar 6;8(3):e58303. doi: 10.1371/journal.pone.0058303 (PMC3590153; doi:10.1371/journal.pone.0058303)
Supplement: Supplement S3 — Children’s Medicines Practices and Preferences Survey for Children. This supplemental item is the survey instrument we used to interview children in Tanzania about their experience in receiving and preferences for children’s medicines. (DOC) [file pone.0058303.s003.doc]

**Supplement S3. Children’s Medicines Practices and Preferences Survey for Children**

1. **Eligibility for Participation in the Children Survey:**
   1. Is interviewee a child between ages 6 and 12, according to parent/caregiver?

Yes  No

- 1. Has informed consent been obtained from a parent/caregiver?  Yes  No
  2. Has assent been obtained from the child?  Yes  No

***If Yes checked for both, then proceed. If not, then either correct or stop. Ideally, children will be interviewed alone. If a parent/caregiver gives consent only if interview is done in his/her presence, then interviewer should instruct parent/caregiver to let the child answer questions.***

1. **Demographic Information:**
   1. Location: Region_______________________
   2. District______________________
   3. Ward (Shehia) ______________________________
   4. Village______________________
   5. Gender of Interviewee:  M  F
   6. Do you know what year were you born?

Yes  No  Don’t Know

2.6.1. If yes, what year were you born? ________

2.6.2 If no, what is your approximate age ___________

- 1. Do you know how much you weigh?

Yes  No  Don’t Know

2.7.1. If yes, how much? _________kg

2.7.2. If yes, how do you know this information?

weighed on a scale

told by health care worker

told by parent/caregiver

guess

Don't remember

- 1. What is the highest grade of school that you completed?

None  Grade 5

Grade 1  Grade 6

Grade 2  Grade 7

Grade 3  Grade 8

Grade 4

- 1. Do you earn any money to bring home?  Yes  No

2.9.1. If Yes, *how*? __________________________________________

1. **Childhood Illnesses –Acute Illness Episode**
   1. Have you ever been sick?  Yes  No  Don’t Know
   2. Can you remember the last time you were sick?  Yes  No

***If No, skip to Section 4***

- - 1. If yes, how long ago was that?

Within last week

Within last month

1-3 months ago

4-6 months ago

7-12 months ago

> 12 months ago

Don’t remember

- 1. What was the name of the illness that you had? ____________________________ ***If you had more than one illness, name and describe the most serious.***
  2. How long did this illness/these illnesses last?____________________

A few days

1-2 weeks

About a month

More than a month

Don’t remember

- 1. What did you do when you first felt sick? ***Check all that apply.***

Told your parent/caregiver

Told a sibling

Told a friend

Rested

Took medicine

3.5.1. If so, what did you take? _________________________

Don’t remember

- 1. What did your parents/caretaker do to make you feel better? ***Check all that apply.***

Took you to health care facility

Gave medicines bought at pharmacy or market

Gave traditional herbal remedy(ies)

Gave special foods or drinks

Nothing

Other, specify________________

Don’t remember

- 1. If your parent/caregiver took you to a health facility, where did you go first? Did you go anywhere else? ***Mark all that apply, beginning with 1 for the place you went first.***

Clinic

Hospital

Pharmacy

Traditional healer

Other, specify_________________

- 1. Were you given any medicines for this illness?

Yes  No  Don’t Know ***If No or Don't Know skip to Question 3.15***

3.8.1. If Yes, what types were they? ***Check all that apply.***

Injections

Pills

Chewable tablets

Syrups

Suppositories (medicines that are inserted into your rectum)

Traditional / herbal remedy(ies)

Other______________________________

- 1. If you took medicines, where did your parent/caregiver get these medicines?

Clinic

Hospital

Pharmacy or market

Traditional healer

A relative or friend

Other__________________

Don’t know

- 1. Does your parent/caretaker had to pay for any of these medicines?

Yes, all  Yes, some  No, none  Don’t Know

- 1. If you had to take pills/tablets by mouth for this illness, how did you take them?

Swallowed the pill or tablet whole

Broke pill or tablet and swallowed

Crushed pill or tablet (whole or pieces) and gave dry powder

Crushed/dissolved whole or piece of pill or tablet and mixed with water

Any other method__________________________________

- - 1. If you took the pill/tablet with water, what was the source of this water? _______________
    2. Was the water boiled before it was mixed with the pill/tablet?  Yes  No  Don’t Remember
    3. If your medicine was crushed before it was given to you, how was it crushed?

Between spoons

Between pieces of paper and rolling a bottle over it

Other (specify) ______________________________

Don’t remember

- 1. Did you ever vomit or spit up these medicines after being given them?

Yes  No  Don’t Know ***If No, skip to Question 3.19***

3.12.1. If Yes, did you vomit or spit up these medicines right away?

Yes  No  Don’t Know

- - 1. If yes, what did your caregiver do when you vomited or spit up the medicine?

Give the medicine again as soon as possible

Skip a dose and give next dose at scheduled time

Stop giving the medicine completely

Nothing

Other ____________________________________

Don't remember

3.12.3. If you did not vomit or spit up these medicines right away, how long after you took the medicines did this happen?

Within a few minutes

Within an hour

More than an hour later

Other ________________________

Don’t remember

- 1. Did you take all medicines that were given to you?  Yes  No  Don’t Know
     1. If no, *why not*? ______________________________
  2. How long did you take these medicines for?

Less 2 weeks

More than 2 weeks

Don’t remember

- 1. How did the medicines taste? _______________________________
  2. Were there medicines you didn’t like?

Yes  No  Don’t Know

3.16.1. If yes, what didn’t you like about them?_________________________

- 1. Were there some medicines that you liked?  Yes  No  Don’t Know

3.17.1 If yes, which ones and *why*?__________________________________________

1. **Childhood Illnesses – Chronic Illnesses Episodes**
   1. Do you have an ongoing illness?  Yes  No  Don’t Know

4.1.1. If Yes, what is the name of this illness? __________________________

***If no or don’t know, skip to question 5.1***

- 1. How did you learn that you have this illness?

From a health care provider

From a laboratory test

From a traditional healer or herbalist

From a family member

Other (specify)___________________________

- 1. When did you learn that you have this illness?

Within last month

1-3 months ago

4-6 months ago

7-12 months ago

> 12 months ago

Don’t know

- 1. Do you take any daily medicines for this illness?

Yes  No  Don’t Know

4.4.1. If yes, what types of medicines do you take? ***Check all that apply.***

Injections

Pills

Chewable tablets

Syrups

Suppositories (medicines that are inserted into rectum)

Traditional / herbal remedies

Other______________________________

***If no, then skip to Section 5.3***

- 1. Where do you get these medicines?

Clinic

Hospital

Pharmacy or market

A relative or friend

Traditional healer or herbalist

Other__________________

- 1. Who gives you these daily medicines?

Health care worker

Parent / caretaker

Other__________________

- 1. Does your parent/caregiver have to pay for these medicines?

Yes, all  Yes, some  No, none  Don’t Know

- 1. If you have to take pills/tablets every day, how do you take them?

Swallowed the pill or tablet whole

Broke pill or tablet and swallowed

Crushed pill or tablet (whole or pieces) and gave dry powder

Crushed/dissolved whole or piece of pill or tablet and mixed with water

4.8.1.What was the source of this water? ________________________

4.8.2. Was the water boiled before it was mixed with the pill/tablet?

Yes  No  Don’t Remember

4.8.3. When mixed with the medicine, is the water:

Hot  Cold  Warm  Don’t remember

- 1. If your medicine was crushed before it was given, how was it crushed?

Between spoons

Between pieces of paper and rolling a bottle over it

Other ______________________________

Don’t remember

- 1. Do you ever vomit or spit up after being given these medicines?

Yes  No  Don’t Know

4.10.1. If Yes, did you vomit or spit up these medicines right away?

Yes  No  Don’t Know

4.10.2. If you did not vomit or spit up these medicines right away, how long after you took the medicines did this happen?

Within a few minutes

Within an hour

More than an hour later

Other ________________________

Don’t remember

4.10.3. If yes, what happens if you vomit after receiving the medicine?

Give the medicine again as soon as possible

Skip a dose and give next dose at scheduled time

Stop giving the medicine completely

Nothing

Other

- 1. In an average week, do you take all the medicines given to you?

Yes  No  Don’t Know

- 1. How do the medicines taste?_____________________________________________
  2. Are there medicines you take that you **don't** like?

Yes  No  Don’t Know

4.13.1. If yes, what **don't** you like about them?_________________________

- 1. Are there some medicines that you **like** to take?  Yes  No  Don’t Know
     1. If yes, which ones do you like? _____________________
     2. *Why*?_____________________________________
  2. Which forms of medicine do you like best? ***Rank in order of preference, using 1 for most favorable.***

Syrups

Pills

Chewable tablets

Injections

Suppositories (medicines inserted into the rectum)

Other

- 1. Why do you like these forms of medicines? _____________________________

1. **Medicines: Use and Access and Expectations**

***If child did not answer the Chronic Illness section, begin with Question 5.1. If the child has answered the Chronic Illness section, begin with Question 5.3.***

- 1. Are you taking medicine now?  Yes  No  Don’t Know
  2. Have you ever had to take medicine for longer than a week?

Yes  No  Don’t Know

- - 1. If yes, was it difficult to take the medicine for a long time?

Yes  No  Don’t Know

5.2.2. If yes, *why*?_____________________

- 1. What do you think are the best types of medicines for children? ______________________
     1. *Why*?_______________________________
  2. What do you think medicines for children should taste like?

Sweet

Bitter

No taste

Other ______________

No preference

Don't know

- 1. Are there some tastes of medicines that you like better than others?

Yes  No  Don’t Know

- - 1. If yes, what tastes? ___________________________________
  1. Do you think children and adults can take the same medicines?

Yes  No  Don’t Know Why or why not?_______________________

- 1. Are there any medicines that you think are bad for children?

Yes  No  Don’t Know

- - 1. Which ones?____________________
    2. *Why*? ___________________________
  1. When you take a medicine, how quickly do you expect to feel better?

Immediately

Within a day

Within a week

Don’t know

Other __________________________

- 1. When you go to a health facility, do you expect to get medicines?

Yes  No  Don’t Know

5.9.1. *Why*? _________________________________________

- 1. How many pills/tablets do you think should take at one time?____
  2. How many pills/tablets do you think you should take in a day?________
  3. Can medicines you take for one illness sometimes be used to treat another illness?

Yes  No  Don’t Know

- - 1. If yes, which medicines can be used for which illnesses?____________________
